# Supplementary material for: Redox Mechanisms Driving Skin Fibroblast-to-Myofibroblast Differentiation
Source: Antioxidants (Basel). 2025 Apr 18;14(4):486. doi: 10.3390/antiox14040486 (PMC12024386; doi:10.3390/antiox14040486)
Supplement: Supplementary file 1 [file antioxidants-14-00486-s001.zip › supplementary table 1, table 2, figure 1--conversion.pdf]

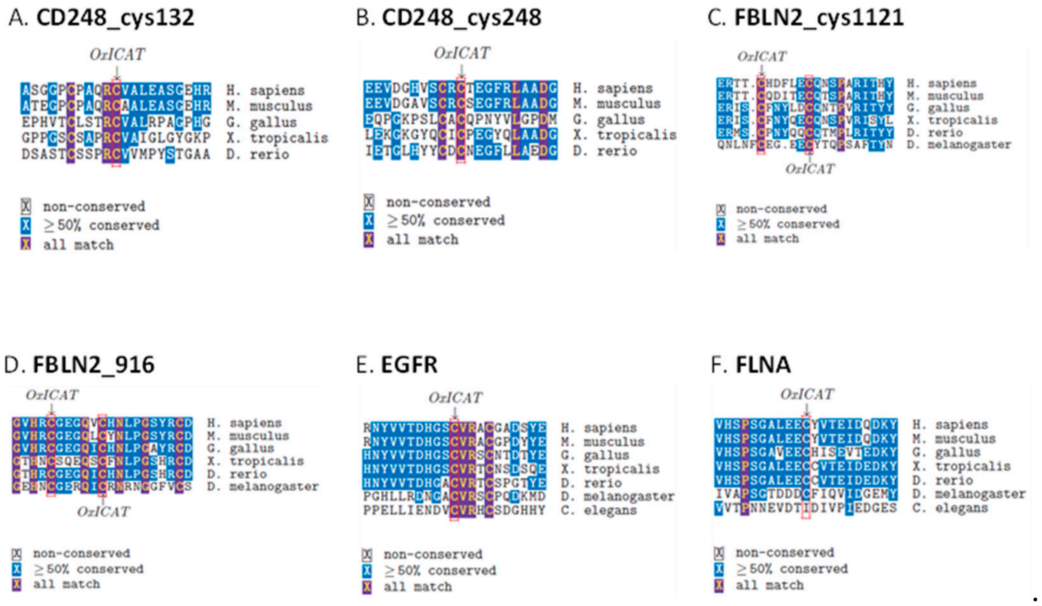

Supplementary Figure 1. Conservation of identified cysteine residues across species: (A, B) endosialin; (C, D) fibulin-2 isoform. E, epidermal growth factor receptor. F, filamin-A.

Supplementary Table 1. Sequence of primers for qPCR.

Gene	Accession	Primer Forward	Primer Reverse
PRDX1	NM_002574.4	CCACGGAGATCATTGCTTTCA	AGGTGTATTGACCCATGCTAGAT
PRDX2	NM_005809.6	GAAGCTGTCGGACTACAAAGG	TCGGTGGGGCACACAAAAG
PRDX3	NM_006793.5	ACTGTGAAGTTGTCGCAGTCT	CACACCGTAGTCTCGGGAAA
PRDX4	NM_006406.2	AGAGGAGTGCCACTTCTACG	GGAAATCTTCGCTTTGCTTAGGT
PRDX5	NM_012094.5	AACAAGGTGAACCTGGCAGA	GGCAGGTGTGTCTTGGAACAT
PRDX6	NM_004905.3	GTTGCCACCCCAGTTGATTG	TGAAGACTCCTTTCGGGAAAAGT
GPX1	NM_000581.4	CAGTCGGTGTATGCCTTCTCG	GAGGGACGCCACATTCTCG
GPX3	NM_002084.5	GAGCTTGCACCATTCGGTCT	GGGTAGGAAGGATCTCTGAGTTC
GPX4	NM_002085.5	GAGGCAAGACCGAAGTAAACTAC	CCGAACTGGTTACACGGGAA
GPX6	NM_182701.1	CAAAGGGGTAACAGGCACCAT	GGCGGCCACATTGACAAAC
GPX7	NM_015696.5	CCCACCACTTTAACGTGCTC	GGCAAAGCTCTCAATCTCCTT
TXNRD1	NM_182729.3	ATATGGCAAGAAGGTGATGGTCC	GGGCTTGTCCTAACAAAGCTG
TXNRD2	NM_006440.5	CGGCTTCGACCAGCAAATG	ACAGGACGGTGTCAAAGGTG
TXNRD3	NM_052883.3	GCTCCTTCAGGAAGATTTGGC	GGGACAACAAAGTCTAGCACCA
TXN	NM_003329.4	GTGAAGCAGATCGAGAGCAAG	CGTGGCTGAGAAGTCAACTACTA
TXN2	NM_012473.4	CTGGTGGCCTGACTGTAACAC	TGACCACTCGGTCTTGAAAGT
TXNDC5	NM_030810.5	CAGAGCCGGAAGTGGAACC	CCACGGAGCGAAGAACTTGAT
TXNIP	NM_006472.6	GGTCTTTAACGACCCTGAAAAGG	ACACGAGTAACTTCACACACCT
GLRX	NM_002064.3	CCCATCAAACAAGGGCTTCTG	CTGCATCCGCCTATACAATCTT
GLRX2	NM_016066.4	TCTTTGGAGAATTTAGCGACGG	CTGGTTTCCATATTCAAGCAGGT
GLRX3	NM_001199868.2	GTCCCTCCTTGTGGTCCATTT	AACTCTGCCATAACTTCGTTCAT
GLRX5	NM_016417.3	CTCCGACAAGGCATTAAAGACT	AACTCGCCATTGAGGTACACT
CAT	NM_001752.4	TGGAGCTGGTAACCCAGTAGG	CCTTTGCCTTGGAGTATTTGGTA
SOD1	NM_000454.5	GGTGGGCCAAAGGATGAAGAG	CCACAAGCCAAACGACTTCC
SOD2	NM_000636.4	GCTCCGGTTTTGGGGTATCTG	GCGTTGATGTGAGGTTCCAG
SOD3	NM_003102.4	ATGCTGGCGCTACTGTGTTC	CTCCGCCGAGTCAGAGTTG

ERO1A	NM_001382464.1	GGCTGGGGATTCTTGTTTGG	AGTAACCACTAACCTGGCAGA
ERO1B	NM_019891.4	TTCTGGATGATTGCTTGTGTGAT	GGTCGCTTCAGATTAACCTTGT
VKORC1	NM_024006.6	CTCGCTCTTTGCCTGACGG	CAGACAGATTGCTTGAGACCAG
QSOX1	NM_002826.5	TGAGAAAGTTTGGTGTCACCG	GGACCTGGATTCCATGAGCAC
QSOX2	NM_181701.4	CTCGTGCAGTTCTACTCGTCG	AGCTCTCGGTCAGGTCCTTT
SELENOF	NM_004261.5	TTTGGGCTACGGTTGTTGTTG	TGCCTCCGATGAAAACTCTGC
NOX1	NM_007052.5	TTAACAGCACGCTGATCCTG	CTGGAGAGAATGGAGGCAAG
NOX2	NM_000397.4	GTACCTGGCTGTGACCCTGT	GGGATTGGGCATTCCTTTAT
NOX3	NM_015718.3	CCT GGA AAC ACG GAT GAG TGA	CCT CCC ATA GAA GGT CTT CTG C
NOX4	NM_016931.5	AACCGAACCAGCTCTCAGAA	TTGACCATTCGGATTTCCAT
NOX5	NM_024505.4	CCA CCA TTG CTC GCT ATG AGT G	GCC TTG AAG GAC TCA TAC AGC C
DUOX1	NM_017434.5	CCAGCAATCATCTATGGGGGC	TGGGGCCGCTGGAACC
DUOX2	NM_014080.5	TCT GAT CCA GCT GCC CAC TT	GCA CCC ACT GTC CTG ACT T
ACTA2	NM_001141945.3	AATACTCTGTCTGGATCGGTGGCT	ACGAGTCAGAGCTTTGGCTAGGAA
Peroxidasin	NM_012293.3	AATCAGAGAGATCCAACCTGGG	AATGCTCCACTAGGTATCCTCTT
FN1	NM_001306132.2	CGGTGGCTGTCAGTCAAAG	AAACCTCGGCTTCCTCCATAA
LOXL2	NM_002318.3	AGGTTGAGAGGATGGCTCGA	ACTGCAAGCACACGGAGGA
POR	NM_001367562.3	GACGACGATGGGAACTTGGA	AGAAGCTCGTACTGGCGAAT

Supplementary Table 2. Accession numbers of proteins highlighting conserved genes across species

Species	EGFR	CD248	FLNA	FBLN2
H. sapiens	NP_005219.2	NP_065137.1	NP_001104026.1	NP_001158507.1
M. musculus	NP_997538.1	NP_473383.1	NP_034357.2	NP_032018.2
G. gallus	NP_990828.2	NP_001383844.1	NP_989905.1	XP_046782597.1
X. tropicalis	XP002939960.2	XP_004913746.1	NP_001361642.1	XP_017949170.2
D. rerio	NP_919405.1	NP_001092698.2	XP_009295045.1	XP_001341277.2
D. melanogaster	NP_476758.1	**NA**	NP_001262658.1	NP_726551.2
C. elegans	CAA93882.3	**NA**	NP_001023550.2	**NA**
